# Supplementary material for: Validation of a Short-Form Version of the Danish Need for Recovery Scale against the Full Scale
Source: Int J Environ Res Public Health. 2019 Jul 2;16(13):2334. doi: 10.3390/ijerph16132334 (PMC6651401; doi:10.3390/ijerph16132334)
Supplement: Supplementary file 1 [file ijerph-16-02334-s001.pdf]

Validation of a short-form Danish version of the Need for Recovery Scale

Supplementary Materials

Contents

Tables

Table S1: Translations of the Need for Recovery items

Table S2: Inter-item correlations

Table S3: Standardized loading values for a single factor analysis

Table S4: Standardized loadings for a dual factor analysis

Figures

Figure S1: Distributions of responses across response categories

Figure S2: Scree plot of the principle components of the Need for Recovery scale

Figure S3: Item information curves for each item

Figure S4: Bland Altman plots showing the relationship between the full- and reduced-scales—exploratory analyses

Figure S5: Bland Altman plots showing the relationship between the full- and reduced-scales—confirmatory analyses

Table S1. The evolution of the Need for Recovery items through the translation from Dutch to English (original questionnaire), from English to Danish (Danish 9-item questionnaire), and back to English again.

Original Questionnaire	Danish 9-Item Questionnaire	Direct Translation
1) I find it hard to relax at the end of a working day.	1) Jeg har svært ved at slappe af efter en arbejdsdag.	I find it hard to relax after a working day.
2) At the end of a working day I am really feeling worn-out.<br>3) My job causes me to feel rather exhausted at the end of a working day.	2) I slutningen af min arbejdsdag er jeg udmattet.	In the end of my work day I am exhausted.
4) Generally speaking, I'm still feeling fresh after supper.	3) Jeg føler mig frisk efter aftensmad.	I feel fresh after dinner.
5) Generally speaking, I am able to relax only on a second day off.	4) Jeg slapper ikke ordentlig af, hvis jeg kun har en dag uden arbejde.	I do not normally relax, if I have only had one day without work.
6) I have trouble concentrating in the hours off after my working day.	5) Jeg har problemer med at koncentrere mig i timerne efter, at jeg er kommet hjem fra arbejde.	I have trouble concentrating in the hours after I come home from work.
7) I find it hard to show interest in other people when I just came home from work.	6) Jeg har svært ved at udvise interesse for andre mennesker, lige når jeg er kommet hjem fra arbejde.	I find it hard to show interest in other people, when I just come home from work.
8) In general, it takes me over an hour to feel fully recovered after work.	7) Det tager mig over en time, før jeg er restitueret /er kommet mig fuldstændigt efter en arbejdsdag.	It takes me over an hour before I am fully recovered/fully improved after a work day.
9) When I get home, people should leave me alone for some time.	8) Når jeg kommer hjem efter arbejde, skal folk lade mig være i et stykke tid.	When I get home after work, people have to leave me alone for a while.
10) After a working day I am often too tired to start other activities.	9) Efter en arbejdsdag er jeg for træt til at begynde andre aktiviteter.	After a workday I am too tired to begin other activities.
11) During the last part of the working day sometimes I cannot optimally perform my job because of fatigue.	-	-

Table S2. The Kendall correlation coefficient between each item of the 9-item Need for Recovery scale. Coefficient values >0.80 could be suggestive of redundant items. Of note—the poor to negligible correlations between items 3 & 4 and almost all other items.

Scale Items	Item 1	Item 2	Item 3	Item 4	Item 5	Item 6	Item 7	Item 8	Item 9
Item 1	1	0.34	−0.19	0.22	0.43	0.31	0.40	0.34	0.25
Item 2	0.34	1	−0.33	0.19	0.41	0.38	0.52	0.37	0.47
Item 3	−0.19	−0.33	1	−0.13	−0.27	−0.28	−0.34	−0.21	−0.33
Item 4	0.22	0.19	−0.13	1	0.25	0.25	0.22	0.18	0.18
Item 5	0.43	0.41	−0.27	0.25	1	0.53	0.50	0.45	0.39
Item 6	0.31	0.38	−0.28	0.25	0.53	1	0.57	0.59	0.45
Item 7	0.40	0.52	−0.34	0.22	0.50	0.57	1	0.54	0.53
Item 8	0.34	0.37	−0.21	0.18	0.45	0.59	0.54	1	0.45
Item 9	0.25	0.47	−0.33	0.18	0.39	0.45	0.53	0.45	1

Table S3. Standardized loadings (pattern matrix is based on the correlation matrix) for a factor analysis assuming only one-factor, based on the scree—plot interpretation.

Scale Items	Factor Loading	
Item 1	0.50	Rotation = 'none'
Item 2	0.62	
Item 3	−0.42	
Item 4	0.32	Number of assumed factors = 1
Item 5	0.68	
Item 6	0.73	
Item 7	0.79	Method = minimum residual
Item 8	0.68	
Item 9	0.65	

Table S4. Standardized loadings for a two factor analysis based on assumptions made using the original questionnaire construct.

Scale Items	Factor Loading 1	Factor Loading 2	
Item 1	0.32	0.22	Rotation = 'oblique'
Item 2	−0.04	0.76	
Item 3	0.00	−0.46	
Item 4	0.23	0.10	Number of assumed factors = 2
Item 5	0.54	0.18	
Item 6	0.83	−0.05	
Item 7	0.43	0.41	Method = minimum residual
Item 8	0.73	−0.01	
Item 9	0.23	0.47	

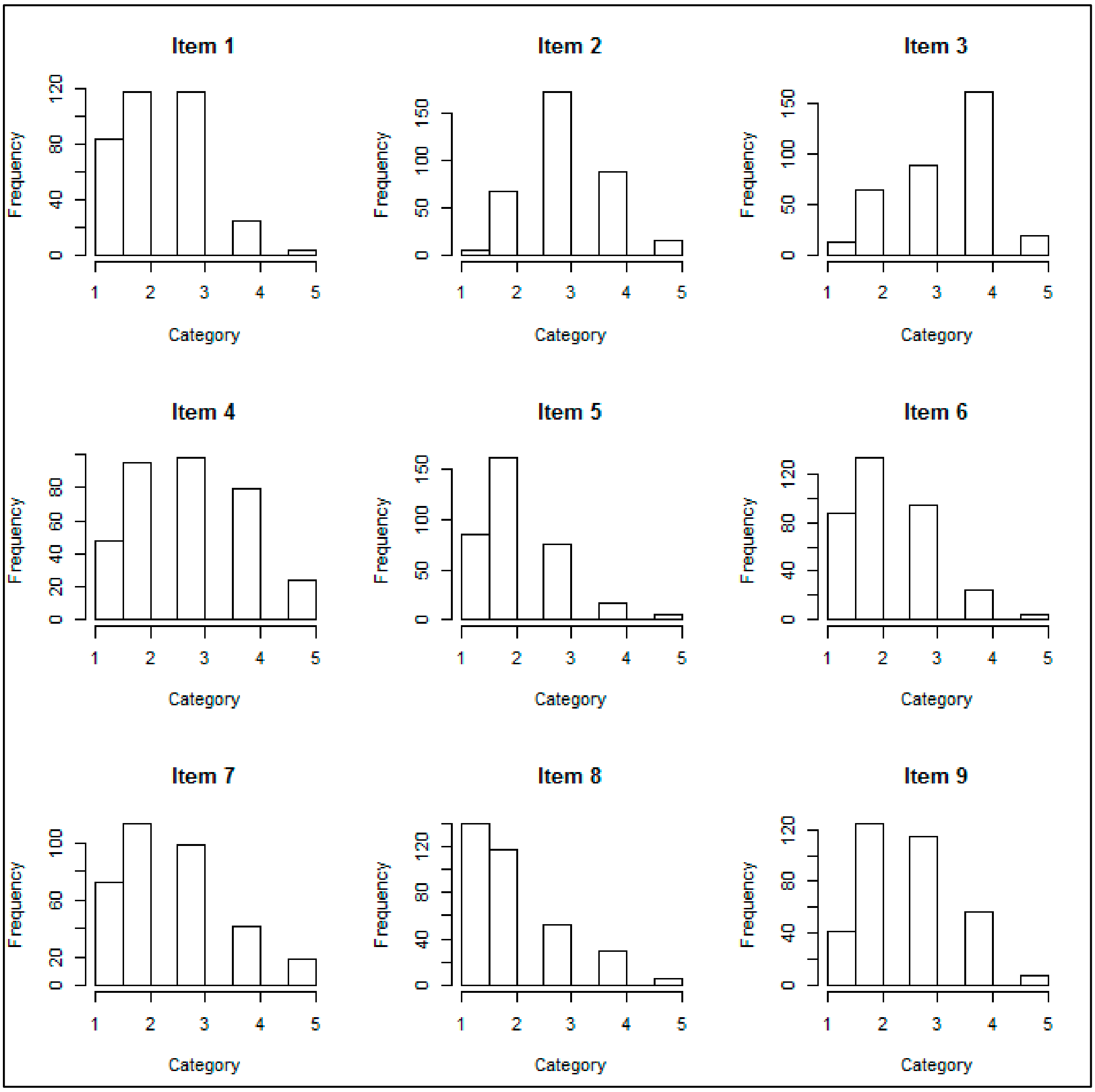

Figure S1. Response distributions for each item of the 9 item scale (detailed in table 1). The x-axis contains the response category (1 to 5) and the y-axis contains the values for the response frequency in actual number of responses.

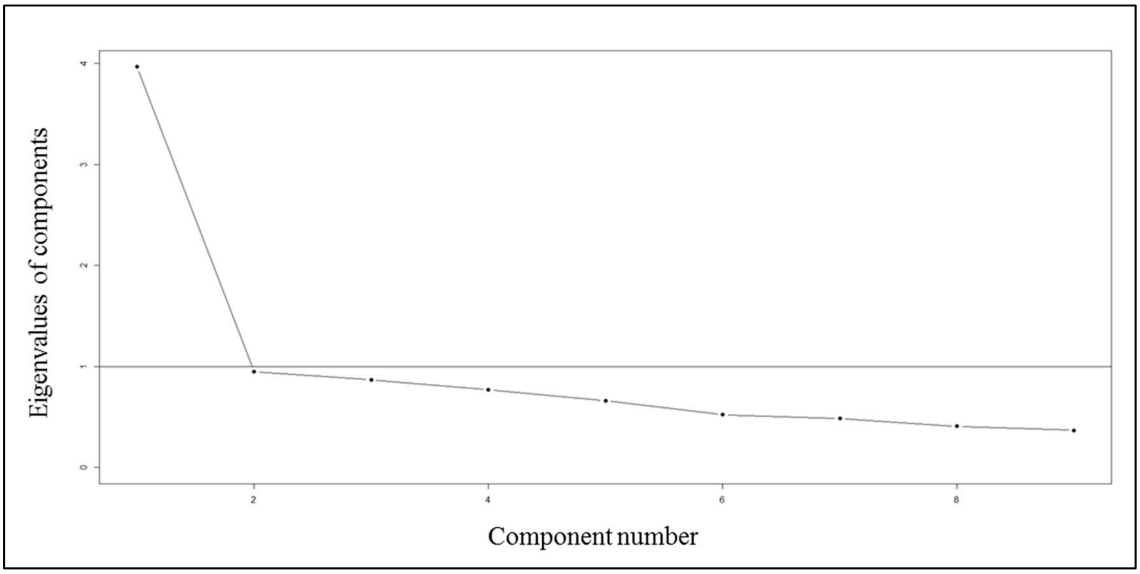

Figure S2. The principal components and eigenvalues of the NFR scale. Eigenvalue threshold is depicted by the horizontal line and is set at a value of one (in line with previous literature). Of note—the second component lies just marginally below this threshold value.

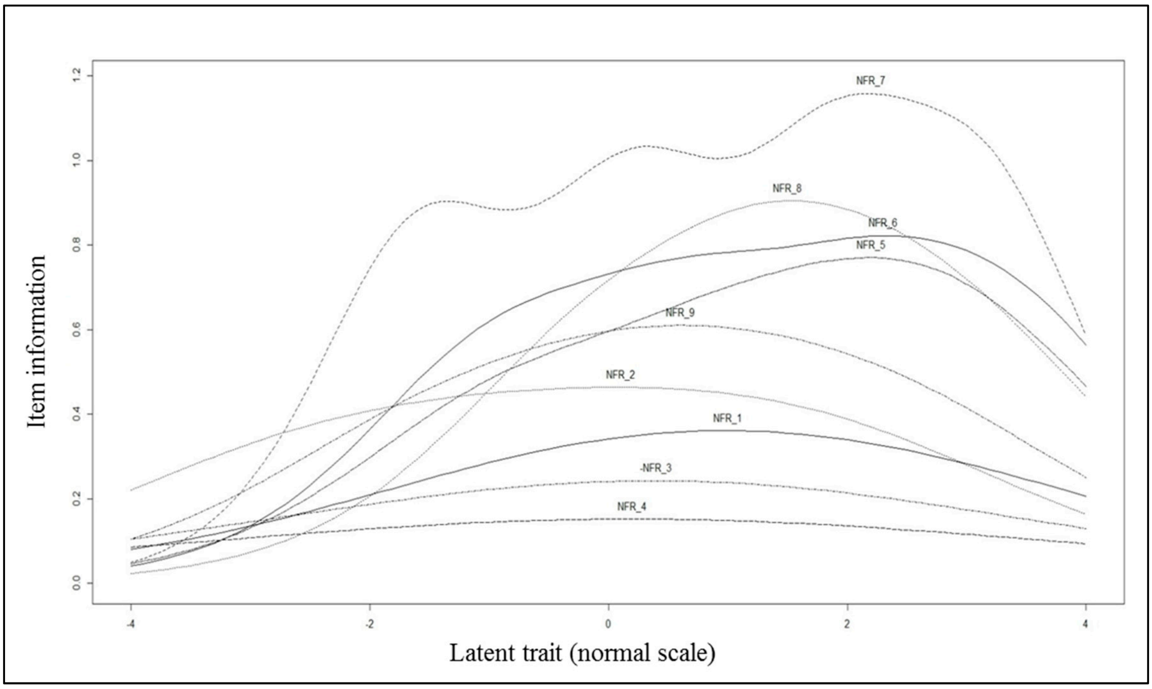

Figure S3. Item Response Theory: Item information scores for each of the 9 items. High slopes indicate areas where an item provides more information for a given level of the underlying trait. The level of information provided by each item is not evenly distributed over all levels of the latent trait.

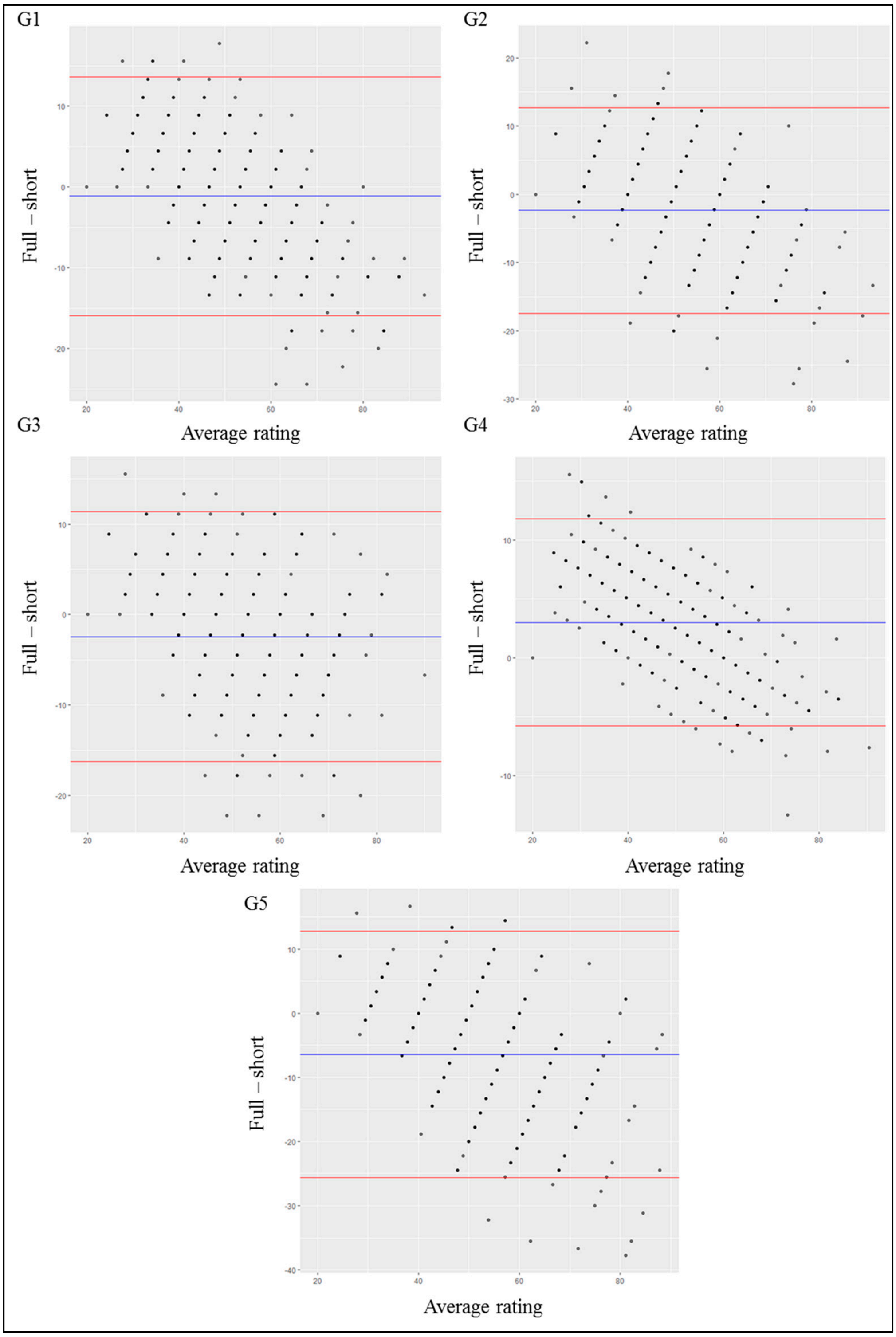

Figure S4. Bland Altman plots showing the agreement between NFR ratings for the full 9-item scale and each short-form version—exploratory analyses. G1 = items 2, 6, & 7; G2 = items 2 & 6; G3 = items 1, 2, & 9; G4 = items 1, 2, & 5–9; G5 = items 2 & 9.

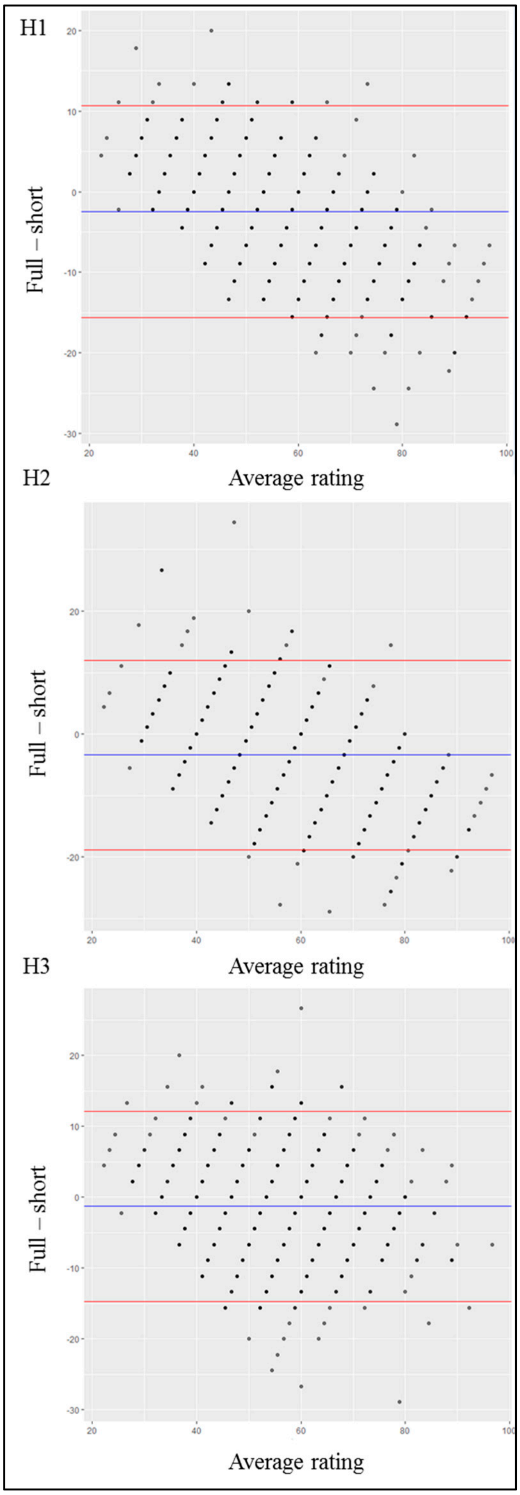

Figure S5. Bland Altman plots showing the agreement between NFR ratings for the full 9-item scale and each short-form version—confirmatory analyses. H1 = items 2, 6, & 7; H2 = items 2 & 6; H3 = items 1, 2, & 9.
